# Phenotypic and Genotypic Detection of Carbapenem Resistance in Urinary Enterobacterales Isolates: A Prospective Cross-Sectional Diagnostic Evaluation Study

**DOI:** 10.7759/cureus.106970

**Published:** 2026-04-13

**Authors:** Pooja P, Raksha Yoganand, Ravi Giriyapur Siddappa, Prathiba Mahadevaiah, Rudresh S M

**Affiliations:** 1 Microbiology, Employee's State Insurance Corporation Medical College and Post-Graduate Institute of Medical Sciences and Research, Bengaluru, IND; 2 Microbiology, All India Institute of Medical Sciences, Bibinagar, IND

**Keywords:** ampc, carba-np test, carbapenemase genes, carbapenem-resistant enterobacteriaceae, enterobacteriaceae, esbl, escherichia coli, klebsiella pneumoniae, polymerase chain reaction, urinary tract infection

## Abstract

Background

Carbapenem-resistant Enterobacteriaceae (CRE)-induced urinary tract infections (UTI) pose increased mortality rates in critically ill patients. Carbapenem resistance is primarily due to the activity of Ambler Classes A, B, and D carbapenemase enzymes. Horizontal transmission of carbapenemase genes has propelled the increase in CRE prevalence. Newer carbapenemase inhibitors do not act against class B enzymes, hence early and precise detection of CRE is essential to advise targeted therapy and manage antimicrobial resistance. In our research, the prevalence of CRE among urinary Enterobacteriaceae isolates was determined, and the diagnostic performance of the Carba-NP test (bioMérieux, Marcy l'Étoile, France) was compared to multiplex polymerase chain reaction (PCR) for carbapenemase detection and the results were analysed. The phenotypic resistance patterns are correlated with particular carbapenemase gene expression.

Materials and methods

An 18-month prospective cross-sectional study was performed, during which 350 Enterobacteriaceae isolates from urine samples were isolated. Disc diffusion was performed according to the Clinical and Laboratory Standards Institute (CLSI) M100-2022 guidelines to screen for carbapenem resistance, and carbapenemase production was phenotypically determined using the Carba-NP test. Multiplex PCR was used to identify prevalent carbapenemase genes (NDM, IMP, KPC, OXA-48-like, VIM).

Results

Among 350 Enterobacteriaceae isolates, 110 were resistant to carbapenems by multiplex PCR. Eighty-one isolates were positive by Carba-NP. Multiplex PCR was positive for most prevalent genes like NDM and OXA-48-like, 44 had both NDM and OXA-48-like, and three had IMP genes. No KPC genes were found. The most common resistant species were Escherichia coli and Klebsiella pneumoniae.

Conclusion

The increased prevalence of CRE among urinary Enterobacteriaceae, which is dominated by NDM and OXA-48-like carbapenemases, highlights the necessity for strong antimicrobial stewardship and infection control measures. The Carba-NP test was 100% specific but had only 73.64% sensitivity compared to PCR, emphasising molecular diagnostics’ critical role in carbapenemase detection. Rapid detection of CRE is required to allow targeted treatment and control the dissemination of antimicrobial resistance in clinical environments.

## Introduction

Enterobacteriaceae is a family that includes Gram-negative rods which produce a range of common infections such as urinary tract infections (UTIs), gastrointestinal infections, skin and soft tissue infections, meningitis, and septicemia. Escherichia coli, Klebsiella pneumoniae, and Enterobacter species are the most common pathogens for UTIs, followed by Citrobacter, Proteus, Morganella, Providencia, and Serratia species [1]. The clinical importance of Enterobacteriaceae is compounded by their high ability to acquire resistance against antimicrobial drugs, making many of the traditional antibiotics ineffective and leading to carbapenem-resistant variants [2]. The chief mechanism of antibiotic resistance in Enterobacteriaceae is the enzymatic production of β-lactamases, hydrolysing enzymes for β-lactam antibiotics. These are categorised into penicillinases, carbapenemases, extended-spectrum β-lactamases (ESBLs), and AmpC-type cephalosporinases, with the most common being ESBLs, which can hydrolyse most penicillins and cephalosporins. As a result, carbapenems have increasingly been used as last-resort drugs in the treatment of infections caused by ESBL-producing bacteria [3].

Carbapenems have wide-spectrum antibacterial activity against Gram-positive, Gram-negative, and anaerobic bacteria. The emergence of carbapenem-resistant Enterobacteriaceae (CRE) is a worldwide critical public health issue. Resistance to carbapenems is due to intrinsic mechanisms involving changes in the porin channels that decrease drug entry or by acquired mechanisms through the production of carbapenemase enzymes and overexpression of ESBLs or AmpC β-lactamases, along with loss of porins [4]. CRE-related UTIs have also become challenging to treat, leading in most cases to treatment failure and higher morbidity, particularly in susceptible populations like those with diabetes, pregnancy, or renal impairment. CRE infection has been linked with increased hospitalisation time, higher healthcare expenses, and increased mortality rates over carbapenem-susceptible infections [2,5].

Noting the emerging threat of CRE, the World Health Organisation (WHO) has listed CRE as a priority antibiotic-resistant pathogen. The primary mechanism of carbapenem resistance is enzymatic inactivation by carbapenemases, which are divided according to the Ambler scheme into Class A, B, and D enzymes. The most disseminated carbapenemases are KPC (Class A), VIM, IMP, and NDM (Class B metallo-β-lactamases) and OXA-48-like enzymes (Class D) [6]. The dissemination of CRE is primarily fueled by the plasmid-mediated horizontal transfer of carbapenemase genes, making infection control a challenge. More importantly, the newer carbapenemase inhibitors are only active against Class A and D enzymes but not against Class B enzymes, emphasising the necessity of rapid and accurate laboratory detection of carbapenemase-producing strains to limit dissemination [7]. Despite continued antimicrobial stewardship and infection control practices, the problem of increasing CRE rates is still unresolved worldwide. Detection of carbapenemase-producing Enterobacteriaceae at an early stage is necessary to direct focused antimicrobial therapy and limit resistance dissemination [8].

Aims and objectives

The present study aimed to explore the occurrence and nature of carbapenem resistance in Enterobacteriaceae isolated from UTIs. The goals were to speciate Enterobacteriaceae isolates from urine cultures, to phenotypically characterise carbapenem resistance by a standard microbiological technique such as the Carba-NP test (bioMérieux, Marcy l'Étoile, France), and to detect the existence of carbapenemase-encoding genes like KPC, NDM, VIM, OXA-48-like, and IMP by multiplex polymerase chain reaction (PCR) assays. With the integration of phenotypic and molecular methods of diagnosis, this research aimed to present a broad overview of resistance mechanisms in carbapenems among urinary isolates, which is significant for directing proper antimicrobial therapy and infection control measures.

## Materials and methods

Study design and duration

This cross-sectional study was conducted in the Department of Microbiology, Employee's State Insurance Corporation (ESIC) Medical College and Post Graduate Institute of Medical Science and Research (PGIMSR), Rajajinagar, Bangalore, for 18 months from October 2022 to April 2024.

Study population and sample size

Three thousand six hundred seventeen (3617) urine samples were received from outpatients and inpatients from different clinical departments. Of these, 350 non-duplicate, single-patient isolates of Enterobacteriaceae were included, considering the sample eligibility criteria. The sample size was calculated using data from a previously conducted Indian study by Paul et al. [9] and an institutional pilot study.

The sample size was calculated based on a pilot study conducted at ESIC Medical College and PGIMSR in 2021, in which the proportion of Enterobacteriaceae was 34.2%. At 95% confidence level, the absolute allowable error of 5%. The estimated sample size is n=345.8 (346). Therefore, at least 350 single-patient Enterobacteriaceae isolates were included in the study.

Methodology

Non-duplicate Enterobacteriaceae isolates alone were included for analysis, which were isolated from clean-catch midstream urine and processed under aseptic conditions. Wet mounts of uncentrifuged urine, revealing a single pus cell per high-power field, were considered relevant. Cultures were carried out using a calibrated 2 mm loop for semi-quantitative inoculation onto 5% sheep blood agar and MacConkey agar. Plates were incubated at 37°C for 18-24 hours, and colony counts were extrapolated as CFU/mL. Significance was considered for bacteriuria alone (>10⁵ CFU/mL). Primary screening was done using colony morphology, Gram staining and battery of standard biochemical reactions like catalase, oxidase, Hugh Leifson's oxidative-fermentative media, nitrate reduction test, methyl red test, acetoin production (VP test), amino acid decarboxylation and dihydrolysis test, indole, mannitol motility test, citrate utilisation test, urea hydrolysis test, triple sugar iron test, and sugar fermentation tests.

Susceptibility testing was done using the Kirby-Bauer disc diffusion method as per Clinical and Laboratory Standards Institute (CLSI) 2022 guidelines. Bacterial inocula were standardised at 0.5 McFarland and plated on Mueller-Hinton agar. Commercial antibiotic discs (HiMedia, Mumbai, India) were used, and plates incubated at 37°C for 18-24 hours. The zone of inhibition was interpreted according to CLSI breakpoints. *Escherichia coli *ATCC 25922was employed as a quality control organism. Antibiotic discs used for testing were carbapenems (imipenem (10µg), meropenem (10 µg)), amikacin (30 µg), amoxyclav (20/10 µg), ampicillin (10 µg), aztreonam (30 µg), cefotaxime (30 µg), cefoxitin (30 µg), ceftazidime (30 µg), ceftazidime/clavulanic acid (30/10 µg), ciprofloxacin (5 µg), cefazolin (30 µg), gentamicin (10 µg), piperacillin tazobactam (100/10 µg), trimethoprim-sulfamethazole (1.25/23.75 µg), tetracycline (30 µg), nitrofurantoin (300 µg), and fosfomycin (200 µg).

**Figure 1:**
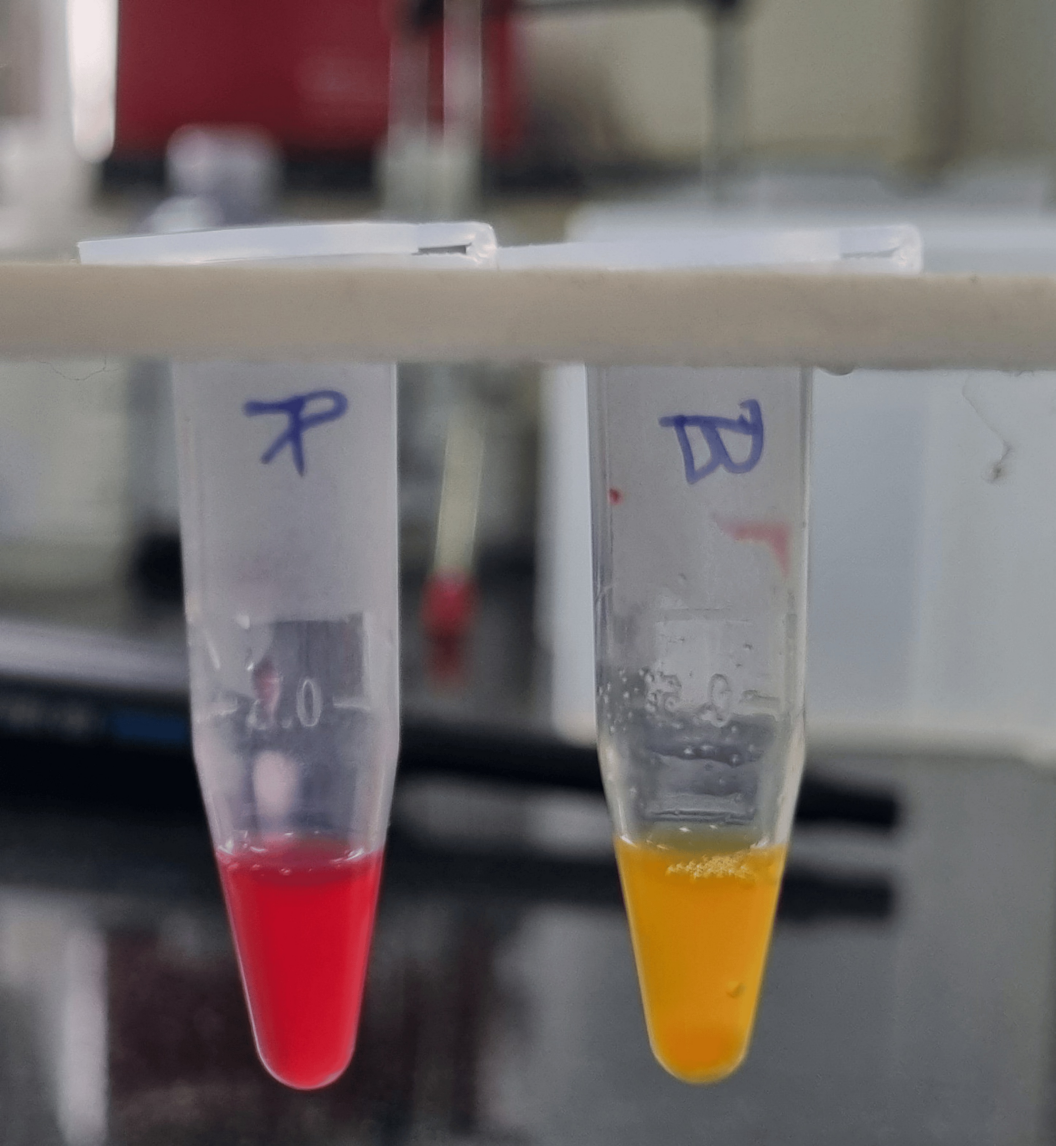
Carba-NP test (bioMérieux, Marcy l'Étoile, France)

Isolates with decreased susceptibility to carbapenems were further analysed for carbapenemase production with the Carba-NP test in Figure 1. Solutions A and B were prepared containing phenol red and imipenem-cilastatin. Two calibrated loops of the bacterial isolate were suspended in 5M NaCl and reacted with reagents. A red to yellow/orange colour change in solution B was a sign of carbapenemase activity. Quality controls consisted of known positive and negative isolates. A strain that gave positive results consistently was taken as a positive control, and another strain that gave consistently negative results was taken as a negative control.

**Figure 2:**
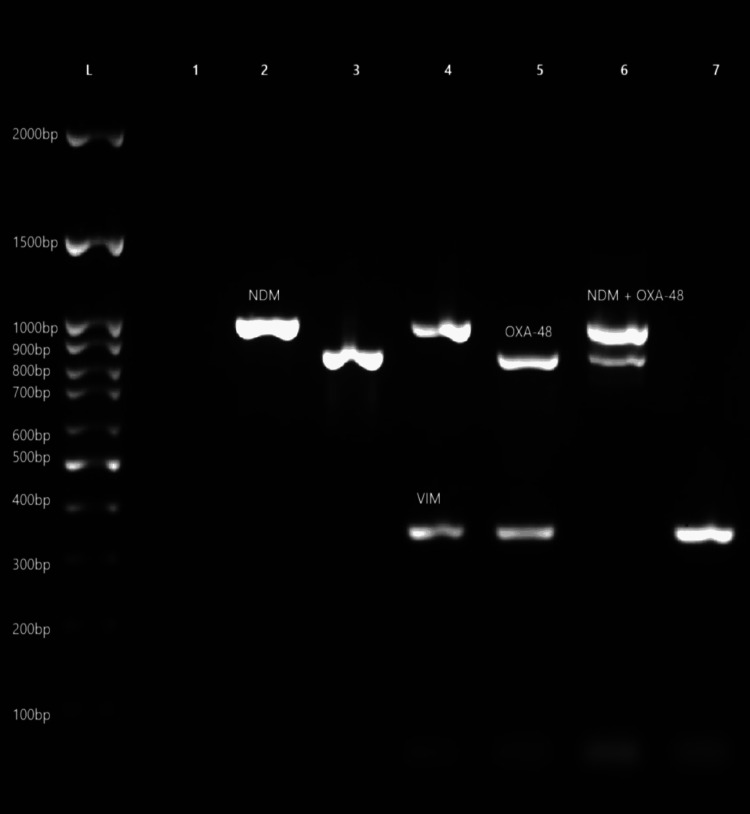
Carbapenemase genes visualized by gel electrophoresis L = Ladder; NDM = New Delhi Metallobetalactamase; OXA-48 = Oxacillinase-48; VIM = Verona Integron-mediated Metallo-β-Lactamase

Multiplex PCR was employed to detect the presence of the listed carbapenemase genes: blaNDM, blaOXA-48-like, blaIMP, blaVIM, and blaKPC in Figure 2. Primers were obtained from earlier published Indian Council of Medical Research (ICMR) antimicrobial resistance (AMR) protocols (Table 1). Extraction of DNA was carried out using the conventional heat lysis method. PCR was conducted in volumes of 25 μL containing 2X PCR Master Mix (Genei, Bangalore, India) using a Bio-Rad thermal cycler (Hercules, CA, USA). Amplification entailed an initial denaturation step at 95°C for 15 minutes, followed by 30 cycles (94°C for 30 seconds, 59°C for 90 seconds, and 72°C for 90 seconds) and a final extension at 72°C for 10 minutes. Amplicons were detected on a 1.5% ethidium bromide-stained agarose gel using the BioRad GelDoc imaging system. Positive control strains that have well-documented gene expression were employed for verification.

**Table 1:** Primers & sequences

Genes	Primers	Product size (bp)
blaIMP	GGAATAGAGTGGCTTAAYTCTC	232
	GGTTTAAYAAAACAACCACC	
blaVIM	GATGGTGTTTGGTCGCATA	390
	CGAATGCGCAGCACCAG	
blaOXA-48 like	TATATTGCATTAAGCAAGGG	800
	CACACAAATACGCGCTAACC	
blaNDM	CACCTCATGTTTGAATTCGCC	984
	CTCTGTCACATCGAAATCGC	
blaKPC	TGTCACTGTATCGCCCGTC	1011
	CTGAGTGCTCTACAGAAAACC	

Statistical analysis

All data were tabulated in Microsoft Excel (Redmond, WA, USA) and processed with Epi Info (CDC, Atlanta, GA, USA) and SPSS version 27.0 (IBM Corp., Armonk, NY, USA). Qualitative variables (sex, age group, sample source, isolate type, resistance pattern, phenotypic and genotypic findings) were represented as frequencies and percentages. The sensitivity, specificity, positive predictive value (PPV), and negative predictive value (NPV) of the Carba-NP test were calculated using MedCalc software and PCR as the gold standard.

## Results

Out of the 350 isolates belonging to the Enterobacteriaceae family, the most frequently isolated organism was Escherichia coli, accounting for 69.4% (243) of the isolates, followed by Klebsiella pneumoniae at 16.6% (58). Other species identified included Citrobacter freundii (3.4%), Enterobacter aerogenes (2.6%), Proteus mirabilis (2%), and smaller proportions of other Enterobacteriaceae (Table 2). A phenotypic test using Carba-NP revealed carbapenemase production among 23.1% (81) of isolates, while 76.9% (269) were negative. By molecular characterisation using PCR, 110 isolates (31.4%) were found to be PCR-positive for carbapenemase genes, while the remaining 240 (68.6%) were negative. Gene-specific analysis of PCR-positive isolates revealed that the most frequently detected gene was NDM+OXA-48 (40%), followed by NDM alone (33.6%), VIM (16.4%), and OXA-48 alone (6.36%). IMP and OXA48+IMP were the least detected, at 2.7% and 0.9%, respectively.

**Table 2:** Distribution of species of Enterobacteriaceae isolated from urine

Isolate	No. of Samples (%)
Escherichia coli	243 (69.4)
Klebsiella pneumoniae	58 (16.6)
Klebsiella oxytoca	5 (1.4)
Citrobacter freundii	12 (3.4)
Citrobacter koseri	6 (1.7)
Enterobacter aerogenes	9 (2.6)
Proteus vulgaris	5 (1.4)
Proteus mirabilis	7 (2.0)
Providentia stuarti	2 (0.6)
Morgonella morganii	3 (0.9)
Total	350 (100)

Phenotypic (Carba-NP) and genotypic (PCR) methods were used to detect carbapenem resistance among the 350 Enterobacteriaceae isolates. As shown in Table 3, 81 isolates were positive by Carba-NP and were also confirmed positive by PCR. However, among the 269 Carba-NP-negative isolates, 29 were found positive on PCR testing, indicating a discrepancy. This yielded a sensitivity of 73.64% and specificity of 100% for Carba-NP when PCR was considered the gold standard. The PPV was 100%, and the NPV was 89.22%, with an overall diagnostic accuracy of 91.71%.

**Table 3:** Carbapenemase genes in PCR-positive isolates (n=110)

	Carba-NP positive, PCR positive		Carba-NP negative, PCR positive	
Genes	No. of Study isolates	Percentage	Number of Isolates	Percentage
IMP	3	2.7	4	13.79
NDM	37	33.6	3	10.3
NDM+OXA 48	44	40.0	2	6.89
OXA 48	7	6.36	17	58.6
OXA48+IMP	1	0.9	2	6.89
VIM	18	16.4	1	3.4

Among the 81 Carba-NP-positive isolates, Escherichia coli was the most prevalent, contributing 61.7% of positive cases. This was followed by Klebsiella pneumoniae (24.7%), with the remainder contributed by other species, including Klebsiella oxytoca, Citrobacter species, and Enterobacter species. Proteus vulgaris and Morganella morganii were not detected in Carba-NP-positive samples. Similarly, PCR-positive isolates were most commonly Escherichia coli, accounting for 70% of the PCR-positive cases, as shown in Tables 4, 5. Klebsiella pneumoniae was the next most frequent (19.1%), followed by minor contributions from Citrobacter, Enterobacter, and other genera. No PCR positivity was seen among Proteus vulgaris and Morganella morganii isolates.

**Table 4:** Diagnostic evaluation of Carba-NP with PCR among the isolates

	PCR positive	PCR negative	Total
Carba-NP positive	81	0	81
Carba-NP negative	29	240	269
Total	110	240	350

**Table 5:** Carba-NP and PCR findings among Enterobacteriaceae isolates

Isolate	Carba-NP		PCR	
	Positive	Negative	Positive	Negative
Escherichia coli	50	193	77	166
Klebsiella pneumoniae	20	38	21	37
Klebsiella oxytoca	2	3	2	3
Citrobacter freundii	3	9	4	8
Citrobacter koseri	2	4	2	4
Enterobacter aerogenes	2	7	2	7
Proteus vulgaris	0	5	0	5
Proteus mirabilis	1	6	1	6
Providencia stuartii	1	1	1	1
Morgonella morganii	0	3	0	3

Among Carba-NP-negative but PCR-positive isolates, gene expression analysis revealed the presence of multiple carbapenemase genes, with the majority being VIM (17 isolates), followed by NDM (four isolates) and OXA-48 (three isolates). This finding suggests that Carba-NP may miss specific genotypes, particularly those producing VIM carbapenemases.

## Discussion

CRE have become a looming worldwide public health threat because of their elevated resistance to a variety of antibiotics, including carbapenems, which are usually last-resort drugs for treatment of severe bacterial infections. Rapid and reliable detection of carbapenemase-producing clinical isolates is important for prompt initiation of proper infection control measures and successful antimicrobial therapy [10]. Among the diagnostic tools in use, the Carba-NP test has emerged as a fast phenotypic test specifically intended to identify carbapenemase production, whereas molecular approaches like multiplex PCR offer confirmatory identification of carbapenemase genes. Knowledge of the diagnostic precision of these tests, their relationship with phenotypic resistance, and the occurrence of multiple carbapenemase genes among clinical isolates is relevant for enhancing patient management and preventing the dissemination of resistant isolates [11,12]. This research sought to evaluate the prevalence of CRE among urinary isolates, contrast the Carba-NP test performance with multiplex PCR, and examine the correlation between phenotypic resistance and carbapenemase gene expression.

Diagnostic performance of the Carba-NP test

The study examined the prevalence of CRE in urinary isolates and compared the performance of the Carba-NP test with the reference standard of multiplex PCR. The Carba-NP test showed a sensitivity of 73.64%, specificity of 100%, and an overall accuracy of 91.71%, which was in tandem with Tijet et al., who obtained 72.5% sensitivity and 100% specificity [13]. Anuradha et al. have also described a reduced sensitivity of 61.76% but a similar specificity of 93.97% [14]. The high positive predictive value and specificity seen in the current study validate the Carba-NP test as a highly sensitive means to confirm carbapenemase production when positive, with the ability for rapid screening that is so critical to clinical microbiology and infection control.

Prevalence and distribution of carbapenemase genes

Molecular PCR characterization indicated that 40% of the isolates harbored both OXA-48-like and NDM genes, 33.6% had NDM only, and 0.9% had OXA-48-like and IMP genes, with no KPC genes present. This is in accordance with the local studies conducted by AlTamimi et al. and Zowawi et al., which demonstrated predominance of OXA-48-like and NDM carbapenemases with infrequent detection of other types like VIM and no KPC [15,16]. Co-existence of NDM and OXA-48-like carbapenemases exacerbates antimicrobial resistance issues and promotes gene transfer to promote greater multidrug resistance.

Silent gene carriage and phenotypic-negative isolates

Notably, 29 phenotypically negative isolates by Carba-NP were PCR-positive for carbapenemase genes such as VIM, NDM, and OXA-48. This silent gene carriage, wherein the presence of resistance genes does not translate to phenotypic expression, has been reported earlier by Emira et al. [17]. Meletis et al. clarified that low-level expression facilitates silent spread of resistance in healthcare settings, underscoring the need for molecular approaches in conjunction with phenotypic testing to guarantee thorough detection of resistance [18]. The findings of this study underscore the intricacy of carbapenemase gene expression alongside the requirement for integration of diagnostic methods.

Correlation of phenotypic resistance patterns with genotype

Phenotypic resistance patterns were very much correlated with carbapenemase gene carriage. The carbapenem-resistant isolates displayed very high resistance to beta-lactams, with a diverse resistance pattern to aminoglycosides and fosfomycin. Garba et al. and Medugu et al. also reported comparable multidrug resistance patterns in E. coli and Klebsiella species, respectively, indicating a reproducible trend across studies [19,20]. The differential susceptibility observed to aminoglycosides and fosfomycin indicates that these drugs are still viable therapeutic alternatives in chosen circumstances, reinforcing the relevance of susceptibility testing as a tool for directing focused therapy.

Limitations

This study’s single-centre design may limit generalizability across diverse healthcare settings. The PCR panel targeted common carbapenemase genes but might have missed emerging or rare variants. Gene expression levels and their impact on resistance phenotypes were not quantitatively assessed, which could further elucidate silent gene carriage mechanisms. Clinical outcome correlations with resistance patterns were not included and warrant future exploration. Future studies should incorporate advanced molecular methods such as whole genome sequencing to detect emerging resistance mechanisms, and integrate clinical outcomes to better correlate diagnostic findings with patient management and prognosis.

## Conclusions

The present study identifies a high rate of occurrence of CRE among urinary isolates, which is primarily mediated by NDM and OXA-48-like carbapenemases. The Carba-NP test possesses high specificity but only moderate sensitivity, confirming its utility as a fast screening assay supplemented by molecular tests for carbapenemase detection. The newer carbapenemase inhibitors like cetazidime-avibactam will act against class A (KPC) and Class D (OXA-48-like) but not against class B (NDM, VIM, IMP). Hence phenotypic resistance correlation with genotypic information is of utmost importance for an accurate diagnosis, informing effective antimicrobial stewardship and infection control to contain the escalating threat posed by multidrug-resistant urinary pathogens.
